# Gamma radiation assisted reduction of graphene oxide in *Persea americana* Mill. seed extract: characterization and oxygen reduction reaction in alkaline and neutral media

**DOI:** 10.1039/d5na01150g

**Published:** 2026-03-10

**Authors:** Nkosingiphile E. Zikalala, Shohreh Azizi, Nomvano Mketo, Ali. A. Zinatizadeh, Touhami Mokrani, Malik M. Maaza

**Affiliations:** a UNESCO-UNISA Africa Chair in Nanosciences & Nanotechnology Laboratory, College of Graduate Studies, University of South Africa Muckleneuk Ridge, P.O. Box 392 Pretoria 0002 South Africa azizis@unisa.ac.za; b Department of Chemistry, College of Science and Engineering and Technology, Florida Science Campus, University of South Africa Johannesburg South Africa; c Environmental Research Canter, Department of Applied Chemistry, Razi University Kermanshah P.O. Box 67144-14971 Iran; d Department of Chemical Engineering, University of South Africa Private Bag X6, Florida Johannesburg 1709 South Africa; e Nanosciences African Network (NANOAFNET)-Materials Research Department, iThemba LABS-National Research Foundation Somerset West, P.O. Box 722 Cape Town 7129 South Africa

## Abstract

Reduced graphene oxide (rGO) exhibits salient properties and thus are applicable in various fields. The major bottleneck to its applications, however, is the long synthesis method that also requires the use of toxic chemicals and high temperatures. Moreover, the surface area of the obtained rGO is often less than that of the starting material, graphene oxide (GO), a phenomenon that compromises its application. In the current study, gamma (γ) irradiation technique where aprotic solvents were substituted with *Persea americana* Mill. seed extract was investigated for its efficacy in reducing GO to rGO by optimizing the total irradiation dose. The successful synthesis of rGO was confirmed with UV-vis spectrophotometry (UV-vis), Fourier Transform Infrared (FTIR) spectroscopy, and Transmission Electron Microscopy (TEM), among others. The X-ray diffraction (XRD) suggested the dominace of reduction on GO with the icrease of the irradiation dosage to 100 kGy (rGO@100). Brunauer–Emmett–Teller (BET) showed that the green reduced GO surface area (60.345 m^2^ g^−1^) is 6 times larger than that of GO (9.586 m^2^ g^−1^). This was confirmed by an enhanced current response on the cyclic voltammetry (CV) of rGO@100 compared to that of GO. The average number of electrons transferred as calculated from the Kotouckey–Levich's (K–L) equation in alkaline and neutral media were 2.04 and 2.26 respectively. This indicates that the electrode (rGO@100/GCE) follows a 2e^−^ pathway mechanisms in both media. The 2e^−^ reaction pathway is also reported when conventional reduction methods are used, therefore the reduction method used in this study is potentially applicable for the development of advanced graphene-based composites for ORR.

## Abstract 2

Gamma ray reduction of GO into rGO in presence of *Persea americana* Mill. seed extract for application in oxygen reduction reaction.

## Graphical abstract

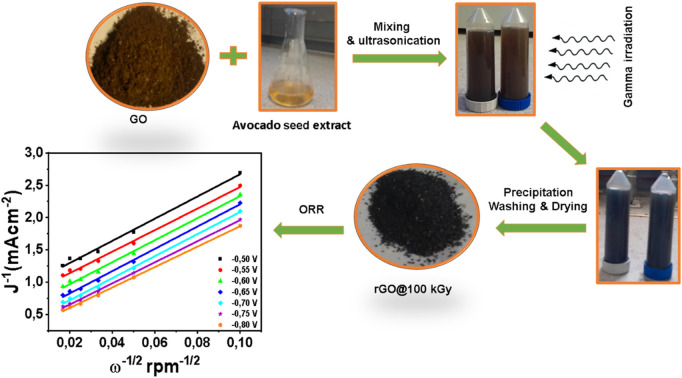

## 1. Introduction

Greener approaches for reducing GO such as electrochemical,^1^ thermal^2^ and employment of various biological reagents as reductants have been explored.^3^ The drawback with the electrochemical GO reduction technique is that it produces small quantities per time and require expensive equipment. The thermal approach demands large amounts of energy, up to 700–1000 °C under a constant flow of nitrogen for 3 h on average. Employing the low temperature (140–180 °C) hydrothermal method requires long reduction time, up to 24 h and produces less graphitized rGO. However, incorporating plant extract as reducing agents has proven to shorten the synthesis time in hydrothermal synthesis to 2 h at even lower temperatures. Therefore many researchers have used various plant extract to reduce GO. However, most reports on green reduced GO are tested on fewer applications such as adsorption studies, degradations pollutants from wastewater and antimicrobial studies.^4–6^ Reports where green rGO has been applied in electrocatalytic oxygen reduction reaction (ORR), hydrogen production and water splitting are scant. With respect to ORR the challenge is that although the mentioned green techniques produce graphene with acceptable electrocatalytic activity, the activity is still lower than that of metal electrocatalysts.^7^ Therefore, there still lies the need to explore other green synthesis procedures that accommodate the use of biocompatible and cheap plant extracts to produce stable and durable graphene-based catalysts with abundant active sites to promote catalytic activity.

One alternative approach is to reduce GO by means of gamma irradiation. Since γ-rays possess the highest energy and shortest wavelength, their penetrating depth through materials is greater due to its zero charge and mass.^8^ For example, the half value layer for pure carbon when a 200 K eV source is used is 2.54 cm.^9^ It is believed that γ-rays achieve a greater degree of uniform reduction, a property that could create more active sites for ORR. Moreover, the process occurs at room temperature, thus significantly cutting energy costs. The γ-radiation reduction method restrains the agglomeration of produced rGO, which is another set-back in rGO production.^10^ In addition, γ-irradiation is clean and safe, with no residual waste making it greener.

Reduction of GO by γ-rays in the presence of water/alcohol medium and nitrogen gas was reported for the first time by Zhang *et al.*^11^ During radiation, reducing agents, hydrogen radicals (H·) and hydrated electrons (e_aq_^−^) as well as oxidizing agents, hydroxyl radical (OH·) and hydrogen peroxide (H_2_O_2_) are produced.^12^ Aprotic solvents such as alcohol scavenge oxidative radicals to prevent further excessive oxidation of GO. However, complete eradication of oxidants is best achieved in an inert atmosphere, which is typically created by purging nitrogen gas into the vials to be irradiated. In one study, alcohol has been replaced with *N*,*N*-dimethylformamide (DMF).^13^ Plants are known to intrinsically possess some reductive potency due to the presence of reducing agents including phytochemicals such as flavonoids, polyphenols, and terpenoids, as well as sugars. Therefore, recently, Atta *et al.* reduced GO in a sonicated mixture of *Hyphaene thebaica* fruit powder and GO solution by γ-irradiating the sample at 80 kGy. Prior, the same group reduced GO *via* the γ-rays technique with natural antioxidants from aloe vera, ginger and a mixture of the two as scavengers of oxidative free radicals. The resultant rGO demonstrated acceptable antioxidant and anti-inflammatory capabilities.^14^ The water–alcohol medium has also been used in the synthesis of rGO composites *via* the γ-irradiation method. For instance, Fe/N-rGO catalyst with varying Fe loadings was prepared by irradiation in a water–ethanol medium at 80 kGy. Calculations from the Koutecky–Levich (K–L) plot indicated that the as prepared catalyst followed a 2e^−^ transfer mechanism that has H_2_O_2_ as an intermediate produced.^15^ In another study, GO supported cobalt oxyhydroxide nanoparticles were synthesized *via* a one-pot γ-radiolysis to catalyze ORR in fuel cells at doses 25, 50 and 100 kGy in water–isopropyl alcohol medium. The optimized catalyst displayed a 4e^−^ transfer mechanism that is most desired for ORR.^16^

*Persea americana* Mill. (avocado) is a berry with one large seed. It is native to Mexico but also abundant in the Sub-Saharan region. A superfood that contains peculiar nutrients, minerals and secondary metabolites therefore it is intensely consumed.^17^ The seed which constitutes between 13–18% of the total fruit's weight is discarded. Relevant to this study, the seed contains natural antioxidants. For instance one study found that the antioxidant capacity of the lipids from the seed extract at 300 lg ml^−1^ dose was 435%, compared to 230% of the standard vitamin C at the same inhibitor concentration.^18^ The antioxidant property stems from phenolic compounds such as flavonoids (catechin, epicatechin, feruloylquinic acid and caffeoylquinic acid) and carotenoids (α-tocopherols and δ-tocopherols).^19^ Epicatechin and catechin have the ability stabilize peroxyl radicals (ROO·), superoxide anions 
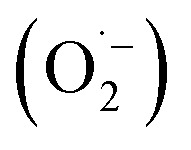
 and hypochlorous reactive species.^20,21^ They achieve this by donating electrons or hydrogen to free radicals as well as reducing and chelating metals. α-Tocopherols also donate hydrogen atoms resulting in their oxidation and loss of antioxidant activity which is regenerated by vitamin C.^22^

Utilizing the seed for the synthesis of nanomaterials as reducing and capping agent not only empowers green synthesis but also adds value to the seed and cleans the environment. According to our knowledge there is no report on the synthesis of nascent rGO or composite synthesized *via* γ-irradiation in the presence of an *P. americana* Mill seed extract in the absence of inert environment. The current study aims, for the first time, to investigate the efficiency of γ-rays assisted reduction of GO where a plant extract (avocado seed) was employed as a natural oxidant scavenger that could possibly eliminate the requirement of aprotic solvents and inert atmospheres during the synthesis of rGO. This is as per the suggested future directions by the authors in.^8^ The structural and electrochemical characterizations for rGO attained at the optimal irradiation dose were performed. Further, the proposed mechanism of reduction was discussed. Finally, the ORR kinetics of the *P. americana* Mill seed extract and γ-ray reduced GO were investigated under alkaline and neutral environments for possible applicability of the resultant rGO as cathode catalysts in alkaline fuel cells or microbial fuel/desalination cells.

## 2. Methodology

### 2.1. Materials

Avocado seeds were obtained from avocados purchased from a local fruits and vegetable shop. Graphite [G, 98%], was purchased from Sigma-Aldrich. Sulfuric acid [H_2_SO_4_, 98%], potassium permanganate [KMnO_4_, AR], hydrogen peroxide [H_2_O_2_, 30–32%], and hydrochloric acid [HCl, 37%] were obtained from ProMark chemicals. Sodium hydroxide [NaOH, AR] and phosphate buffered saline [0.1 M PBS] were purchased from Radchem PTY LTD. Deionized (DI) water [18 MΩ] was obtained from Milli-Q Water Systems (Millipore Corp. Bedford, MA, USA, deionized (DI).

### 2.2. Synthesis of graphene oxide

Graphene oxide was synthesized as per the improved Hummers' method as reported by Goswami *et al.*^23^ In a typical reaction, 5 g of graphite was placed in 1 L conical flask to which 115 mL H_2_SO_4_ was added and magnetically stirred at 350 rpm for 45 min at room temperature (*RT*). The reaction flask was then placed on an ice bath before a slow addition of 15 g KMnO_4_. The stirring was allowed for 45 min before removing the ice, followed by raising the temperature to 45 °C with continued stirring for 30 min. DI water (200 mL) was added while the stirring progressed for further 2 h at 45 °C. To terminate the reaction, a mixture of 100 mL DI water and 40 mL H_2_O_2_ was added slowly into the reaction followed by sonication for 30 min. The mixture was then left to stand over night to decant the excess supernatant (acid and water) before washing with 10% HCl. This was followed by neutralizing using DI water until the pH was ∼6. The resultant thick brown slurry was dried in a vacuum oven at 60 °C for 12 h to obtain film sheets of GO that were crushed and stored in airtight vials for characterization and application.

### 2.3. Gamma irradiation treatment of graphene oxide

The brown skin (testa) of the seed was removed before grating into rough powder. The rough powder was subsequently ground into a fine powder using a pestle and mortar. Thereafter, the *P. americana* Mill seed extract was prepared by adding 1 g finely ground powder into 200 mL deionized water to make 5 mg mL^−1^ concentration as reported by.^24^ The seed extract solution was heated in an oil bath at 60 °C for 90 min followed by cooling to RT and subsequent centrifuging at 4500 rpm for 10 min. The supernatant whose pH was measured to be 4.12 was kept at 4 °C for further use. A 10 mL solution of (5 mg mL^−1^) *P. americana* Mill seed extract and 200 mg GO were poured into a clear tube and ultrasonicated for 1 h before exposed to irradiation for a total dose of 25, 50, 75 and 100 kGy respectively. These were labelled rGO@25-rGO@100 for ease of reference. The γ-ray source was panoramic 1 MCi ^60^C at iThemba Laboratories for Accelerator Based Science (LABS). The required time to irradiate the samples was determined using eqn (1). The attained samples were washed several times with distilled water and dried at 45 °C for 24 h.1
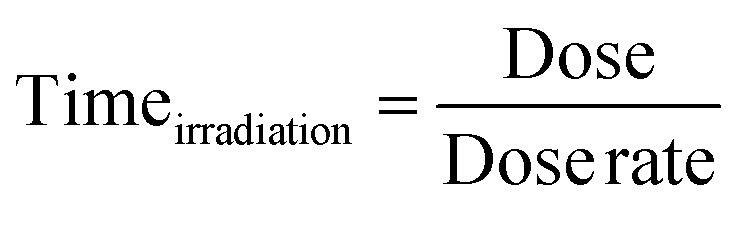


### 2.4. Characterizations

#### 2.4.1. Structural and morphological

Optical absorbance measurements were carried out in the 200–800 nm wavelength range using UV-vis spectrophotometer (UV-vis: Ocean optics-USA, Maya 2000 Pro and DH-2000-BAL UV-vis-NIR light source). The surface functional groups of the samples were examined *via* Fourier Transform Infrared spectroscopy (FTIR) using a PerkinElmer ATR-FTIR Microscope Spotlight 400 (Waltham, Massachusetts, USA). For transmittance measurements, each sample was scanned in the range 600–4000 cm^−1^. The structural defects on the samples were determined by Raman spectroscopy (A Witec Alpha 300 R confocal) with a ND: YAG laser excitation source (532 nm) in the range 200–2000 cm^−1^. A Rikagu Smartlab diffractometer (Tokyo, Japan) was used to perform X-ray diffraction spectroscopy (XRD). The diffractograms were gathered continuously in the 2*θ* range of 10–70° at a scan rate of 5.08 deg min^−1^. To image the surface morphology of the materials at low resolution, a scanning electron microscopy (SEM), (Jeol SEM IT 300, Tokyo Japan) and for higher resolution Particle size and morphology were investigated using a Jeol JEM 2100 series (Kyoto, Japan) transmission electron microscopy (TEM) operated at 200 kV. The surface area, pore size and volume for GO and rGO were probed using Brunauer–Emmett–Teller (BET) Micromeritics TriStar II analyzer (Micromeritics USA) using N_2_ as the probing gas at 77 K.

#### 2.4.2. Electrochemical

PGSTAT 302N electrochemical workstation (powered by Nova software version 2.1.6) was used for cyclic voltammetry (CV) and electrochemical impedance spectroscopy (EIS) studies. The electrochemical investigations were carried out using a standard three-electrode system, where a platinum (Pt) wire served as the counter electrode, Ag/AgCl (sat. KCl) electrode as the reference electrode and a glassy carbon electrode (GCE) with a geometric surface area of 0.0706 cm^2^ was the working electrode. Prior to testing, the working electrodes surfaces were cleaned with Milli-Q water and polished with slurries of alumina powder (down to 0.06 mm). To remove any adsorbed material, the electrodes were also sonicated in ethanol for 10 min followed by sonication in Milli-Q water for another 10 min. For the CV and EIS measurements the GCE was modified using drop-dry method where 5 µL (1 mg mL^−1^ GO or rGO in DMF) aliquots were deposited at the electrode surface and kept in an oven at 60 °C for 10 min. Characterizations (CV and EIS) of the bare GCE and modified GCE electrodes, carried out in 0.001 M [Fe(CN_6_)]^3−^/[Fe(CN_6_)]^4−^ solution containing 0.1 M KCl. [Fe(CN)_6_]^3−/4−^ as a redox probe that exhibits 1-electron reversible process.

### 2.5. Oxygen reduction reaction studies

To carry out the CV and EIS studies under oxygen saturated media, the GCE electrode with a surface area of 0.07 cm^2^ was cleaned and modified as previously mentioned. For the CV studies, the potential was swept from −0.25 to 0.8 V. For the rotating disk electrode (RDE) studies, the working electrode was GCE with a surface area of 0.1963 cm^2^. Freshly prepared 10 µL (1 mg mL^−1^) catalyst ink in DMF were drop-casted on polished surface followed by drying at 60 °C for 10 min. The ORR studies were performed in alkaline conditions by using 0.1 M NaOH as the electrolyte and in neutral media where 0.1 M PBS served as the electrolyte.

## 3. Results and discussions

### 3.1. UV-vis

**Fig. 1:**
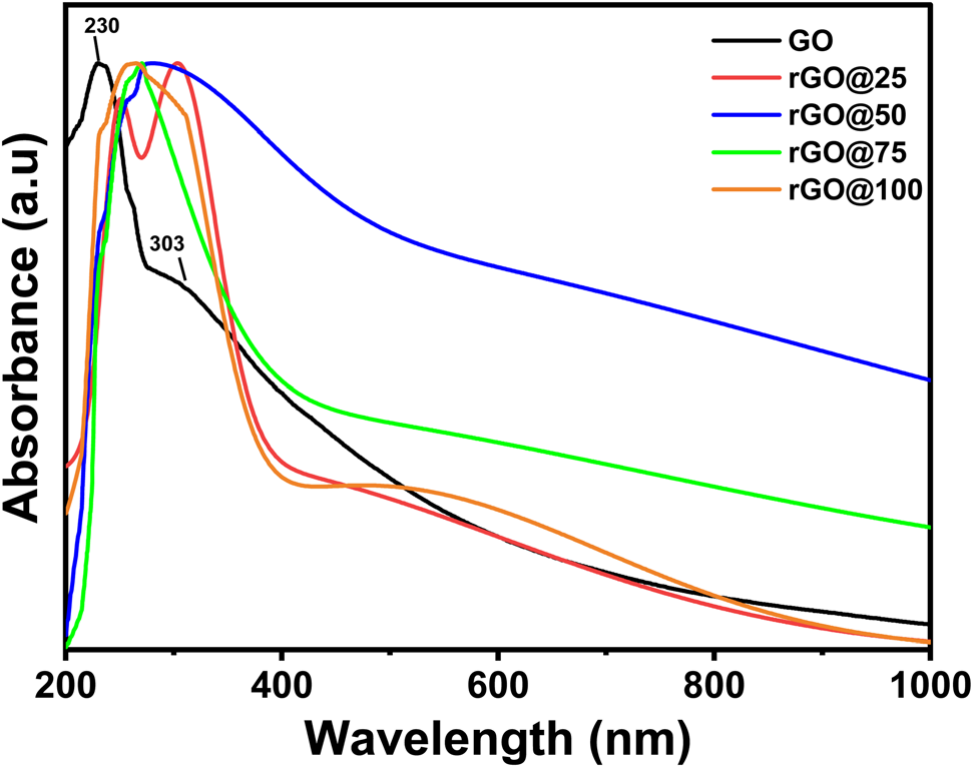
The normalized absorbance spectra of GO gamma radiated at various doses.

The UV-vis for rGOs obtained at different γ-irradiation doses in the presence of *P. americana* Mill seed extract is shown Fig. 1. The UV-vis spectra for GO have a sharp absorption peak at 230 nm, that emanates from the π → π* electronic excitation of the C=C matrix. The shoulder peak at 302 nm is due to *n* → π* electronic transitions in the oxygen-containing functional groups (–COOH, –CHO, and C–O–C). Optical transitions between π and π* states in the sp^2^ clusters that remain in the structure of rGO after its oxidation also result in this peak.^25^ Exposing the samples to even lower dose (rGO@25) caused a red-shift of the π → π* peak to 251 nm.^26^ Interestingly, the 303 nm shoulder peak (*n* → π*) intensified. This was presumed to be due to the attachment of some oxygenated groups present in the extract on the surface of GO and a possible further oxidation of GO by the oxidizing species that are formed as a result of water radiolysis since the GO/*P. americana* Mill seed solution was not purged with nitrogen as commonly done in traditional γ-ray GO reduction studies.^27^ The rGO@50's *n* → π* transition peak was eliminated and a red-shift of the π → π* transition peak to 275 nm was apparent. The red-shift was due to enhanced conjugation of C=C bonds in its skeleton^28^ as a consequence of a strong interaction between the strong reductive e_eq_^−^ with a reduction potential of −2.9 V and some interaction of the seed extract phytochemicals with the π–π bonds in GO.^29^ Further increase of the total dose led to a concurrent narrowing of the peaks bandwidth and a blue-shift from to 261 nm in rGO@100.

### 3.2. Fourier transform infrared analysis

**Fig. 2:**
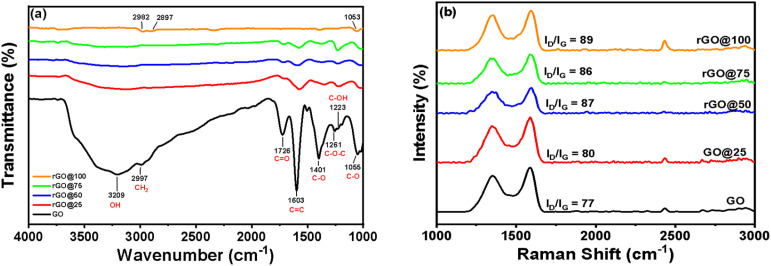
(a) FTIR (b) Raman spectra of pristine GO and rGO reduced at different gamma radiation doses.^56^

FTIR spectroscopy is an effective method for identifying various functional groups present on the surface of materials. This technique provides semi-quantitative information about the chemical composition in that the amount of the present functional groups is directly correlated to the peak intensities.^30^Fig. 2a presents the FTIR of GO that exhibits a strong and broad peak at 3209 cm^−1^ corresponding to O–H stretching vibration modes of –COOH and C–OH functional groups.^31^ The absorption band at 2997 cm^−1^ is due to C–H groups whereas the weak absorption signal at 1726 cm^−1^ corresponds to stretching vibration of the carboxyl group. The strong 1603 cm^−1^ band is attributable to the C=C stretching vibration of aromatic rings and O–H bending vibration of water molecules^32^ while the 1401 cm^−1^ corresponds to C–OH vibration of the carboxyl group. The peak at 1261 cm^−1^ is characteristic of the C–O–C vibration in epoxy whereas the signal at 1223 cm^−1^ corresponds to C–OH stretch of alcohol group.^33^ The signal from 1055 cm^−1^ comes from the C–O vibration of an alkoxy.^34^ The FTIR of γ-irradiated rGO at different doses shows that all the oxygenated functional groups intensities are significantly reduced compared to that of GO while the C–OH, OH and C–O–C completely disappeared at rGO@100. Based on the FTIR results, the optimal γ-ray dose for reducing GO in *P. americana* Mill seed extract is 100 kGy.

Fig. 2b represents the Raman spectra of the samples irradiated at various doses. The D and G peak positions were determined after baseline subtraction. The D and G bands originate from structural defect (disorder-induced mode) and first order scattering from the *E*_2g_ phenom of sp^2^ carbon bonding, respectively. The intensity ratio of the D and G bands (*I*_D_/*I*_G_) correlates to the size of sp^2^ hybridized graphene in the γ reduced GO.^35^ The *I*_D_/*I*_G_ ratios for rGOs irradiated at 25, 50, 75, 100 kGy were 0.80, 0.87, 0.86, and 0.89 respectively. The increase of the *I*_D_/*I*_G_ ratios at lower γ-radiation dose (rGO@25-rGO@50) was attributed to faster formation of sp^2^ domains than the accumulation of defects (moderate defect regime) while the subsequent decrease at rGO@75 is ascribable to non-monotonic behavior of rGO system.^36^ At 75 kGy (high defect regime), more defects such as vacancies formed leading to amorphization of the carbon structure, a phenomenon that reduces the *I*_D_/*I*_G_ ratio. A similar observation was reported by Ji *et al.* who noticed that, during irradiation, sites that were previously occupied by oxygen containing groups become graphitized while new vacancies are formed on the edges and surfaces the graphene sheets.^37^ As the dose increased to 100 kGy the material seemingly experienced a rearranging of the carbon structure that caused the ratio to increase again. It is noteworthy that the *I*_D_/*I*_G_ increased only by ∼9% between GO@25 and GO@100 suggesting that the number of defect sites remained almost the same at all the irradiation doses. The same trend was observed by^38^ when reducing free standing GO films *via* γ-irradiation in gaseous phase.

### 3.3. X-ray diffraction spectroscopic analysis

**Fig. 3:**
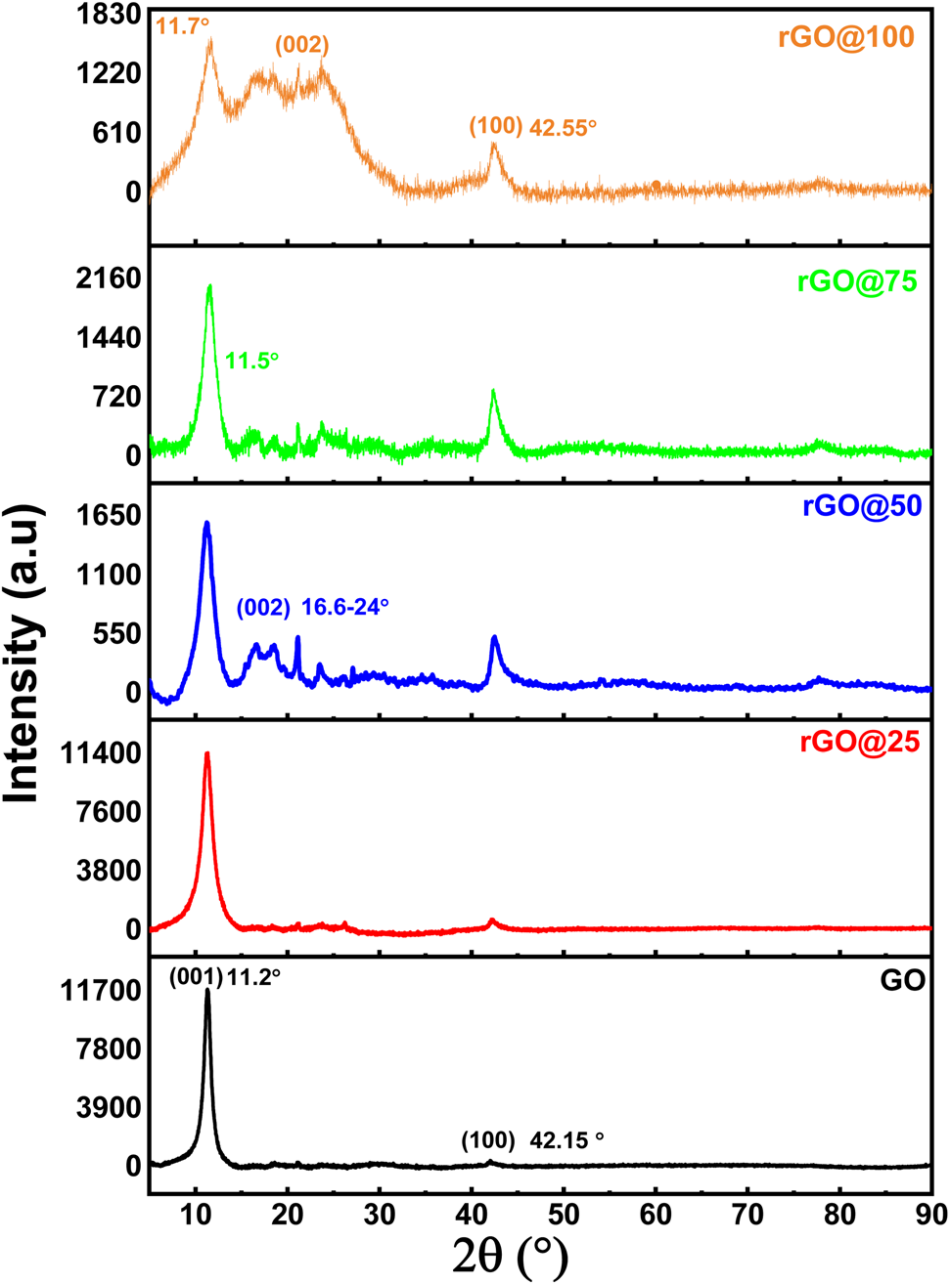
XRD results for pristine GO and irradiated GO at different doses.

The XRD spectra was measured in the range of 2*θ* = 5° to 90° (Fig. 3). The difractogram for GO shows a (001) reflection that corresponds to stacked, oxidized layers at 2*θ* = 11.30° (*d*_001_ = 0.782 nm) with an intensity of 11 831 counts. After γ-irradiation treatment at low dose (rGO@25), the intensity of this peak reduced by 249 counts which was accompanied by the emergence of a broad peak at 2*θ* = 16.6–24° (002) that corresponds to graphitic regions. At the highest irradiation (rGO@100), the (001) shifted to 11.7 (*d*_001_ = 0.756 nm) and the intensity significantly reduced by ∼85% to 1588 counts. The (002) also increased remarkedly by ∼78% from 260 in GO to 1161 intensity counts in rGO@100. The reduction of the intensity counts for the (001) plane suggests removal of some oxidized groups while the enhancement of the (002) plane with the irradiation dose points to formation of more sp^2^ domains in the structure. The presence of a substantial (001) even at the highest dosage could be due to incomplete removal of oxygenated groups since samples were not purged with nitrogen gas. The broad (002) reflection in rGO@100 proposes poor regulation along the stacking direction and also confirms that the material mostly consists of free graphene sheets.^39^ Its *d*_002_ was 0.381 nm a value closer to 0.341 of graphite and thus confirms reduction. The (100) observed at 2*θ* = 42.15° in GO shifted to 2*θ* = 42.55° in rGO@100. The persistence of this diffraction plane suggests that the hexagonal carbon lattice of graphene was preserved during the reduction process.

The Bragg's equation was applied to (001) and (002) reflections to determine the interlayer distance (*d*) between the sheets. Eqn (2) (Scherrer's relationship) was applied on (001) and (002) reflections to calculate the average height of stacking layers (*L*_c_) and the average diameter of stacking layers (*L*_a_).2
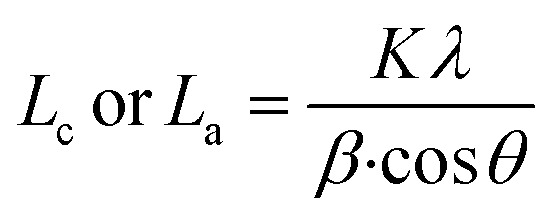
where *λ* is the radiation wavelength (0.15406 nm), *θ* is the diffraction peak position of (001/002), *β* is the peak's full width at half-maximum in radians; and *K* is a constant (0.9 for *L*_c_ and 1.84 for *L*_a_).

The number of graphene layers was calculated using eqn (3),^40^3
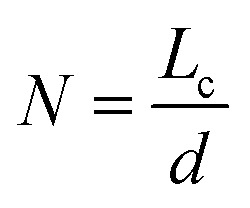


The results were presented in Table 1.

**Table 1:** Structural parameters GO and rGO@100 kGy from the XRD patterns

Material	Peak (001)					Peak (002)					Peak (100)		
	2*θ* (°)	FWHM (nm)	*L*_c_ (nm)	*d* (nm)	*n*	2*θ* (°)	FWHM (nm)	*L*_c_ (nm)	*d* (nm)	*n*	2*θ* (°)	FWHM (nm)	*L*_a_ (nm)
GO	11.30	1.17	6.83	0.79	8–9	—	—	—	—	—	42.2	0.87	20
rGO@100	11.48	4.34	1.84	0.755	2–3	23.30	7.92	1.02	0.381	2–3	42.6	2.03	8.6

GO was found to consist of 8–9 layers in a stacking with an average diameter of 20 nm and a height of ∼7 nm. The rGO@100 sample on the other hand consists of 2–3 layers in stack with an average diameter of ∼9 nm by and height of ∼2 nm.

### 3.4. Scanning and transmission electron microscopy

**Fig. 4:**
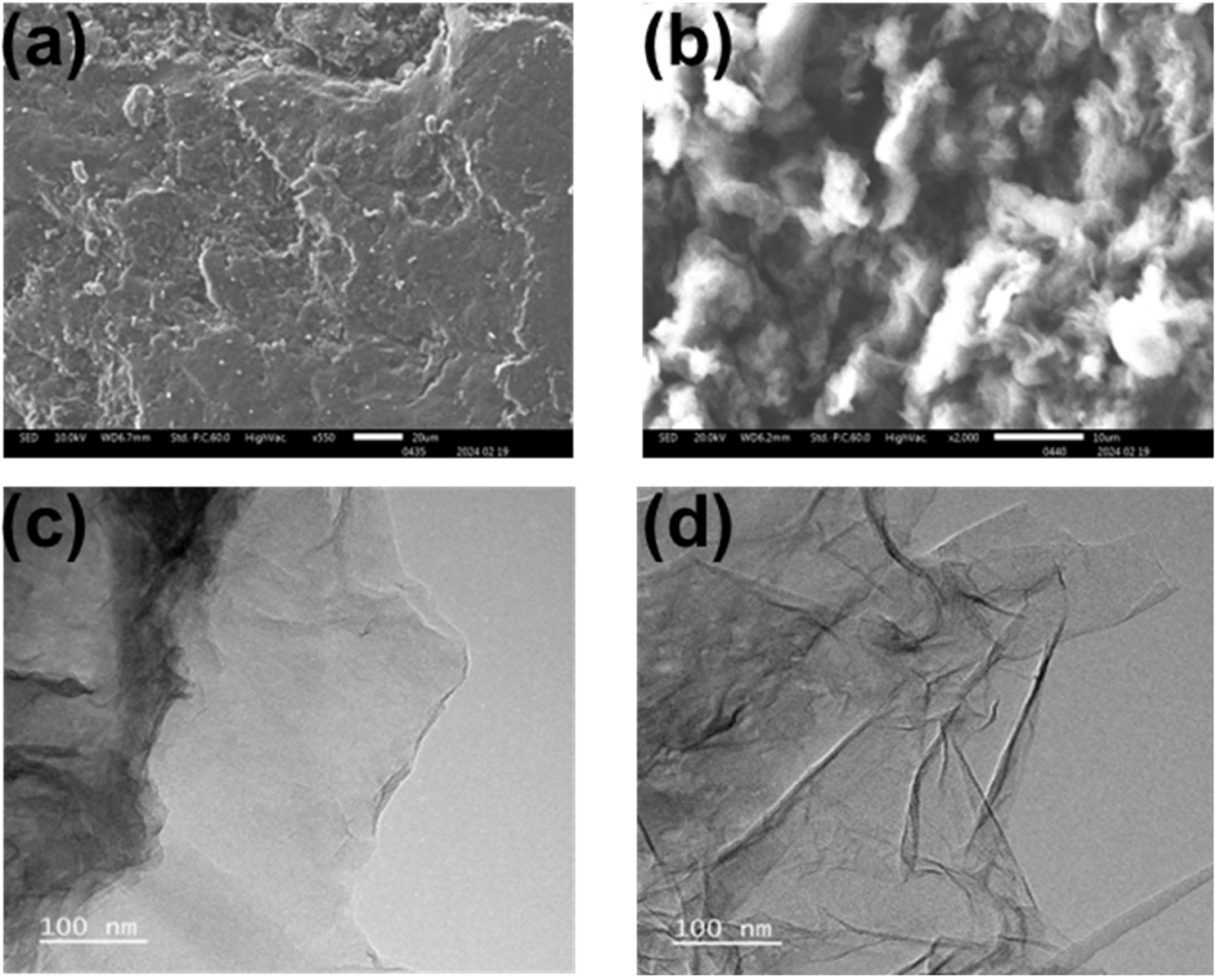
SEM images of (a) GO and (b) rGO@100. TEM micrographs for (c) GO and (d) rGO@100.

The morphology of GO, and rGO@100 were examined by scanning electron microscopy (SEM) and TEM as indicated in Fig. 4. Fig. 4a shows stacked GO sheets morphology whose layered structure appears to be peeling off. This agrees with the XRD. The peeling layered structure is as a result of imbalance of graphite that occurs after oxidation and exfoliation into mono or multilayer GO.^41^ After γ-irradiation at GO@100, free-standing rGO sheets with wrinkles were apparent (Fig. 4b). The wrinkles hamper further aggregation caused by Van der Waals interactions. The TEM for GO in Fig. 4c exhibits a flat surface with very few wrinkles while the GO@100 sample consist of translucent wrinkled and folded regions (Fig. 4d). The wavelike appearance is due to the elimination of the oxygenated functional groups on the surface of the material.^42^

### 3.5. Surface area and porosity

**Fig. 5:**
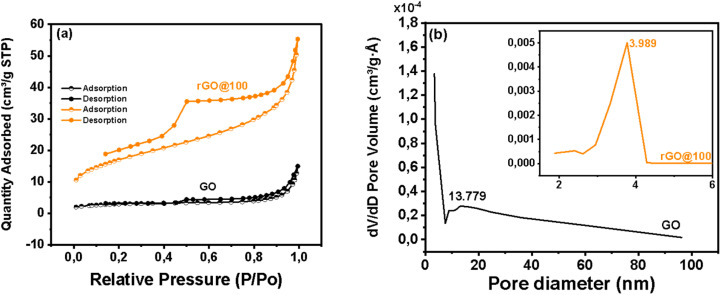
(a) N_2_ sorption isotherm and (b) pore diameter of the as-synthesized GO and rGO@100.

The surface area for GO and rGO@100 was determined *via* BET. The N_2_ adsorption isothermal plots are presented in Fig. 5. Both the plots display hysteresis loops at higher relative pressures that are a distinct shape of type IV adsorption which propose that the adsorption of N_2_ is multilayered on the open surface and pores of the mesoporous nanomaterials.^43^ According to IUPAC classification, the hysteresis loops for the samples, are classified as H4 (Fig. 5a) which infers that the pores are slit-like due to interconnectedness of the graphene sheets. The specific BET surface area (*S*_BET_) for GO was found to be 9.586 m^2^ g^−1^ compared to 60.345 m^2^ g^−1^ of rGO@100. Although the surface area values for GO and rGO@100 are smaller than some values reported in literature^44,45^ and as shown in Table 2, the *S*_BET_ for GO is comparable to other samples prepared *via* the Hummers' method^46^ and better than others.^47^ In as much as the obtained *S*_BET_ for rGO@100 is also lower than some rGO's presented in Table 2, it is noted that during the reduction, the surface was enhanced 6.3 times. The degree of surface area enhancement upon reduction in this study is higher than some rGO counterparts reduced *via* various methods including thermal (Table 2). It is however difficult to conclude on the best reduction method that enhances *S*_BET_ as the thermal approach for instance may significantly improve the surface area (∼114 times) as seen in^47^ but can also raise it only moderately (∼3 times). In some instances, reducing GO to rGO leads to even a significant decline in the *S*_BET._^45,48^Fig. 5b shows the pore size distribution curves obtained using the Barrett–Joyner–Halenda (BJH) analysis. The average pore diameters and single point pore volumes were 13.779 nm and 0.011100 cm^3^ g^−1^ respectively for GO. The corresponding values were 3.989 nm and 0.060182 cm^3^ g^−1^ for rGO@100. A detailed summary of the surface area analysis is found in Table S1. The pore diameters confirm the mesoporous nature of the materials while the enhanced pore volume is in agreement with the surface area. An enhanced surface area and mesoporous nature structure are ideal characteristic for an ORR catalyst and explains the improved current response in the CV for rGO@100 compared to GO in later sections.

**Table 2:** Presentation of *S*_BET_ for GO and rGO@100 synthesized *via* different reduction methods and surface area enhancement after the reduction

Reduction method	Reducing agent	Parameters	GO *S*_BET_ (m^2^ g^−1^)	RGO *S*_BET_ (m^2^ g^−1^)	rGO/GO	Ref.
Plant mediated	*P. americana* Mill. and gamma irradiation	RT/100 kGy	9.59	60.35	6.3	This work
Plant mediated	*Hibiscus Sabdarriffa* L, hydrothermal	100 °C	318.06	506.69	1.6	44
Green (biomolecule)	Ascorbic acid and NaOH	90 °C	141.26	29.50	0.2	45
Thermal	Heat	600 °C	141.26	368.58	2.6	45
Hydrothermal	Water and NaOH	185 °C	141.56	383.16	2.7	45
Chemical	Acetone	120 °C	8	30.00	3.8	46
Thermal	Heat	500 °C	4	455.00	113.8	47
Chemical	Hydrazine hydrate	90 °C	304.43	93.12	0.31	48

### 3.6. Cyclic voltammetry

**Fig. 6:**
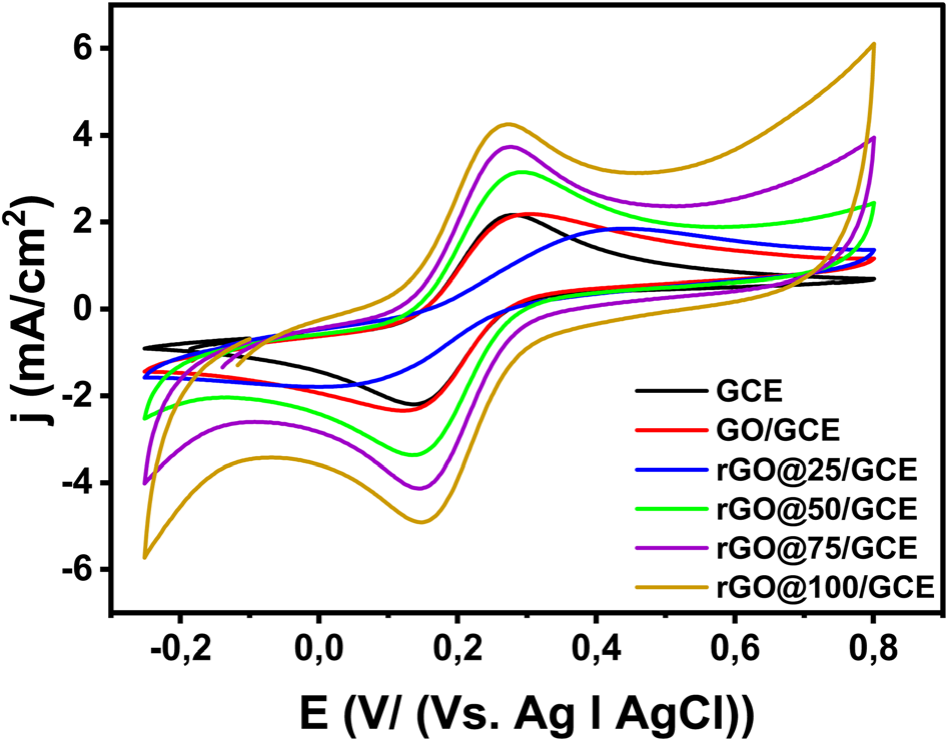
Cyclic voltammogram of GCE, GO/GCE and GCE@ various doses. Scan rate = 100 mV s^−1^, in 0.1 M KCl containing 1 mM [Fe(CN)_6_]^3−/4−^ solution.

The CV is an invaluable tool for investigating electron transfer-initiated chemical reactions.^49^Fig. 6 presents CV evolutions of the bare GCE, GO/GCE, rGO@25/GCE, rGO@50/GCE rGO@75/GCE and rGO@100/GCE electrodes recorded in 1 mM [Fe(CN_6_)]^3−/4−^ solution containing 0.1 M KCl at 100 m Vs^−1^ from −0.2 to 1 V *vs.* Ag|AgCl. The redox peak potential separation (Δ*E*_p_) for the bare electrode (GCE) is 131 mV. The Δ*E*_p_ for GO/GCE increased to 160 mV accompanied by a slight broadening of current densities (Table 3) indicating a slower electron transfer due to some hindrance by GO adsorption.^50^ The voltammogram of the rGO@25/GCE electrode exhibits a significant reduction of the peak currents as well as an increase of Δ*E*_p_ to 375 mV. This result affirms the enhancement of the 303 nm peak in the UV-vis spectra (Fig. 1) that was likely due to oxidation from some phytochemicals from the plant extract on the catalyst surface. The oxidized phytochemicals blocked the active sites and slowed down the electron transfer kinetics. Further increment of the radiation dose led to narrowing of the Δ*E*_p_ to 145 mV, 122 mV, and 115 mV for rGO@50/GCE, rGO@75/GCE, and rGO@100/GCE respectively as indicated in Table 3. The reduction in Δ*E*_p_ is accompanied by a gradual increment of the peak currents implying an enhancement of electron transfer. The enhanced electron transfer of rGO@100/GCE *versus* nascent GO is attributable to the large surface area as per the BET results and increased conductivity of rGO as a result of oxygen removal. The reversible nature of the electrodes is confirmed by the *I*_pa_/*I*_pc_ value that is very close to 1 (ref. 51) (Table 3).

**Table 3:** A summary of CV data for the different surfaces in 1 mM [Fe(CN)_6_]^3−/4−^

Electrode	Δ*E*_p_ [Fe(CN)_6_]^3−/4−^ (mV)	*I*_pa_ *j* (mA cm^−2^)	*I*_pc_ *j* (mA cm^−2^)	*I*_pa_/*I*_pc_
GCE	131	1.52	1.47	1.03
GO/GCE	160	1.52	−1.46	1.04
rGO@25/GCE	375	1.41	−1.38	1.02
rGO@50/GCE	145	1.93	−1.92	1.01
rGO@75/GCE	122	2.14	−2.12	1.01
rGO@100/GCE	115	2.12	−2.28	0.93

### 3.7. Electrochemical impedance spectroscopy

**Fig. 7:**
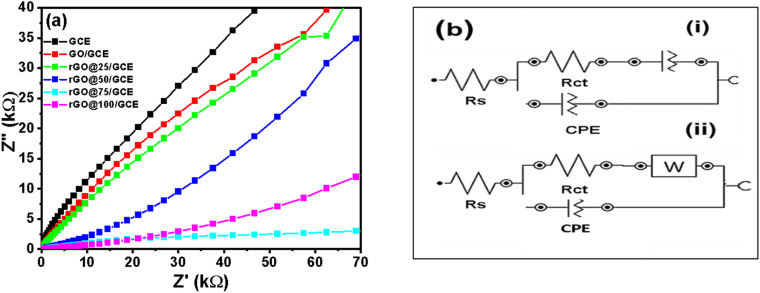
(a) Comparative Nyquist plots (b) Randles circuit used for the various electrodes studied in 0.1 M KCl containing 1 mM [Fe(CN)_6_]^3−/4−^.

To complement the CV data, the electrochemical impedance spectroscopy (EIS) analysis was carried out to study the heterogeneous electron transfer kinetics at these electrodes. The EIS measurements were performed with Autolab Frequency Response Analyzer (FRA) software between 10^5^ Hz and 0.1 Hz using 0.01 V rms sinusoidal modulation in 1 mM of [Fe(CN)_6_]^3−/4−^ containing 0.1 M KCl and the Nyquist plots are presented in Fig. 7a. The experimental data were satisfactorily fitted with the modified Randles electrical equivalent circuit (Fig. 7b(i)), commonly used for modelling a redox-active monolayer on an electrode surface. The fitting parameters of a Nyquist plots involved the electrolyte resistance (*R*_s_), electron transfer resistance (*R*_ct_), and constant phase element (CPE_1_) replaced the true double layer capacitance (*C*_dl_). CPE_2_ was, however, replaced with the Warburg-type impedance (*Z*_w_) for rGO@75/GCE (Fig. 7b(ii)). A high *R*_ct_ implies low electrode conductivity or high resistance.^52^ The *R*_ct_ values were, 24.7 kΩ, 36.1 kΩ, 40.2 kΩ, 2.77 kΩ, 5.64 kΩ and 1.07 kΩ for GCE, GO/GCE, rGO@25/GCE, rGO@50/GCE, rGO@75/GCE and rGO@100/GCE respectively (Table 4). Upon modifying the electrode with GO the *R*_ct_ increased further for the rGO@25/GCE before reducing consistently with the γ-radiation dosage. The observed *R*_ct_ trend corroborates that of Δ*E*_p_. It can be concluded that irradiation of rGO@100 significantly improved the conductivity and hence the electron transfer of the electrodes. The apparent heterogenous electron transfer rate constant (*k*_app_) values of the electrodes in Table 4 were obtained from eqn (4).4
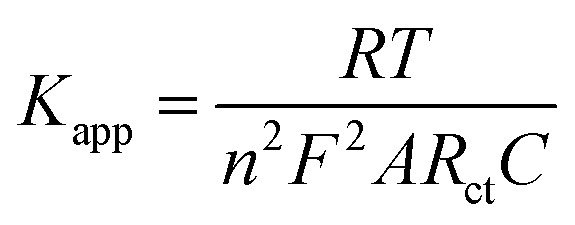
where *n* is the number of electrons transferred, *F* is the Faraday constant, *A* is the geometric area of the electrode, *R* is the ideal gas constant, *T* is the absolute temperature (K) and *C* is the concentration of the [Fe(CN)_6_]^3−/4−^ (in mol cm^−3^). The *k*_app_ values decrease as follows: rGO@100/GCE > rGO@50/GCE > rGO@75/GCE > GCE > and GCE/GO and rGO@25/GCE. The results indicate an easier electron transfer between the redox probe and the GCE at the rGO@100/GCE compared to the other electrodes. The *K*_app_ agrees with both the Δ*E*_p_ and the *R*_ct_ values. Bode plots and discussion that support these finding are presented Section S2 and Fig. S2.

**Table 4:** Impedance parameters obtained for the various electrodes studied in 1 mM [Fe(CN)_6_]^3−/4−^ in 0.1 M KCl solution

Electrode	*R*_ct_/kΩ	*n*_1_	10^3^*k*_app_/c ms^−1^
GCE	24.7	0.81	0.34
GO/GCE	36.1	0.78	0.16
rGO@25/GCE	40.2	0.77	0.16
rGO@50/GCE	2.77	0.73	1.36
rGO@75/GCE	5.64	0.71	0.68
rGO@100/GCE	1.07	0.78	3.50

### 3.8. Proposed mechanism of GO reduction

Since the samples were suspended in an aqueous *P. americana* Mill seed extract, upon exposure to γ-rays, the water molecules broke down into reducing agents, H⋅ and e_aq_^−^ as well as oxidizing species, OH⋅ and H_2_O_2_.^12^ Other oxidants may have arose from the extract as well. Based on the UV-vis and XRD results, at low dosages, reduction of GO was insignificant probably because the dosage was insufficient to produce adequate reducing species to scavenge oxidizing agents and that further deoxygenated GO. As the irradiation dose increased however, phenolic compounds like flavanoinds and carotenoids potentially in addition to H· and e_aq_^−^ from water radiolysis reduced the oxidizing species and further deoxygenated GO. α-Tocopherol could donate electrons to decompose H_2_O_2_, and scavenge peroxyl (ROO·), OH· and alkoxyl radicals (RO·) as well as ·O_2_ originating from the radiolysis of water and possibly the extract.^19–21,53^ The deoxygenation of GO could have occurred in different ways, such as OH· being add to carbonyl or split epoxy bridges to form C–H bonds. This is confirmed by the FTIR (Fig. 2a) for rGO@100 when compared with unirradiated GO where the carbonyl (C=O) at 1726 cm^−1^ that is associated to –COOH group at the edge of GO nanosheets becomes significantly weakened while the C–H absorption bands at 2982 and 2897 cm^−1^ became stronger. Alternatively, reactions might take hydroxyl groups away to form C=C. While this aspect is not clearly depicted in the FTIR, the (002) diffraction plane which is associated with restoration of the sp^2^ bonding of the graphitic structure in GO@100 (Fig. 3) depicts improved intensity and broadness. Moreover, the red-shift of the π → π* transition peak in the UV-vis spectrum at higher dose (Fig. 1) is ascribed to improved conjugation of C=C.^28^ The e_aq_^−^ might have reacted with carbonyl and carboxyl groups to form ketyl radicals, which can recombine with the reductive radicals from the phytochemical's molecules.^54^ Briefly, oxygen-containing groups were eliminated thus restoring conjugation of C=C groups consequent to interactions between reductive species and oxygen-containing groups on GO.^55^

### 3.9. Electrocatalytic reduction of oxygen in alkaline media

**Fig. 8:**
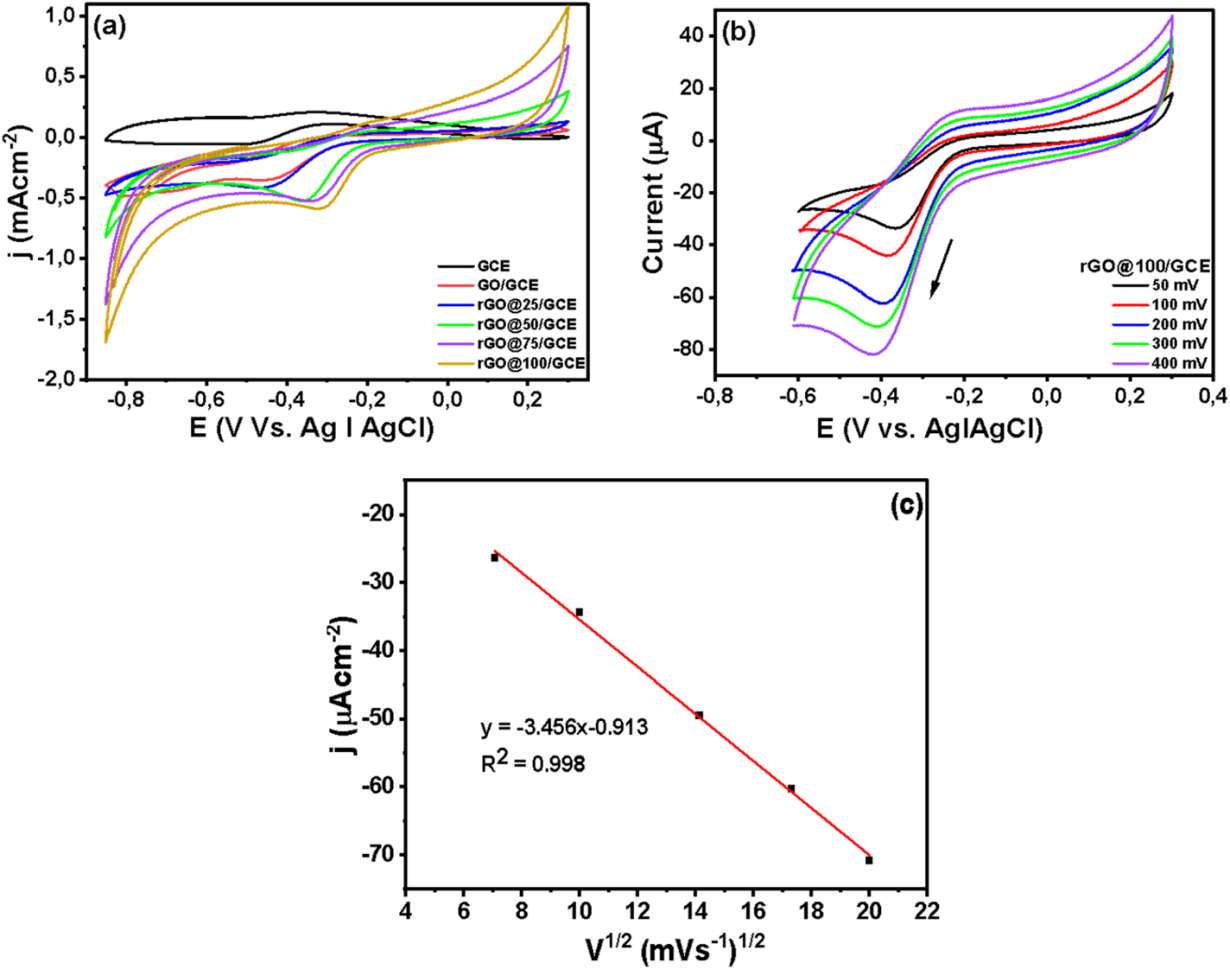
(a) Comparative cyclic voltammograms of the various electrodes in oxygen saturated 0.1 M NaOH solution at 100 mv s^−1^, (b) cyclic voltametric evolutions at different scan rates, (c) plots of peak current *vs.* square root of scan rate.

Unmodified rGO is known to drive the ORR through a 2-electron pathway that produce H_2_O_2_ as a mid-step before producing H_2_O.^56^ The ORR activity of the rGOs was tested in 0.1 M oxygen saturated NaOH solution. Fig. 8a shows that rGO@100/GCE exhibits a better catalytic activity towards ORR in terms of the onset potential (−0.19 V) and catalytic current response of approximately −1.69 mA cm^−2^ compared to the rest of electrodes that displayed more negative onset potentials accompanied by lower current densities. The ORR peak positions were GCE (−0.49 V), GO/GCE (−0.44 V), rGO@25/GCE (−0.45 V), rGO@50/GCE (−0.35 V), rGO@75/GCE (−0.32 V), and rGO@100/GCE (−0.31 V). The effect of varying the scan rate (50–400 mV s^−1^) on the GO@100/GCE electrode was also interrogated (Fig. 8b). The peak current against the square root of scan rate plot shows that the peak potentials shift to more negative potential in a linear form with an increase of the scan rate (Fig. 8c) an indication of an irreversible diffusion-controlled reaction.^57^

#### 3.9.1. Rotating disc electrode studies in alkaline medium

**Fig. 9:**
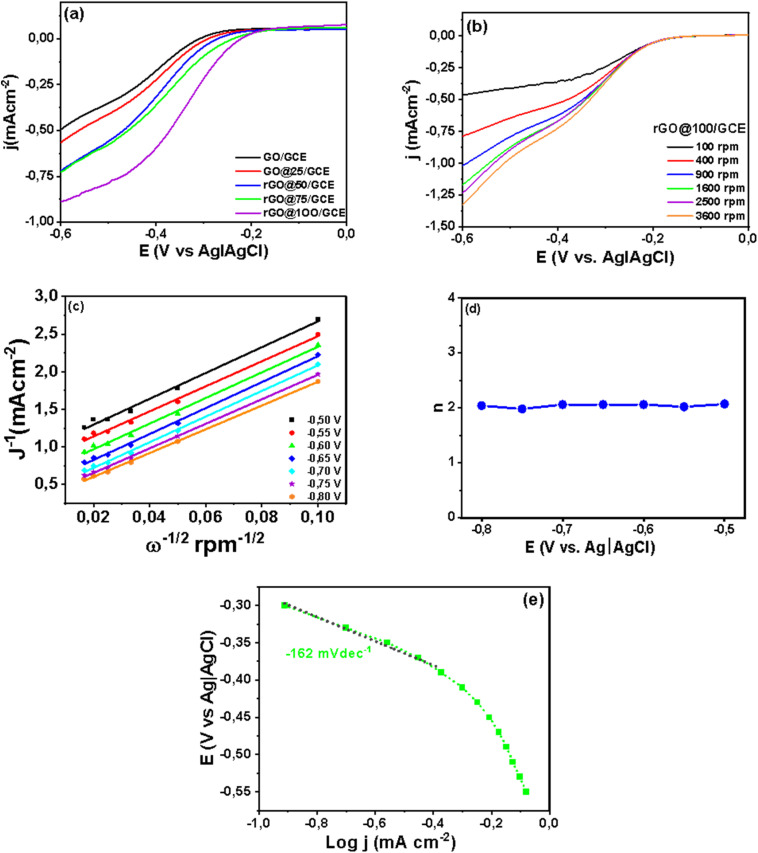
RDE polarization curves (a) GO irradiated with different total doses at 500 rpm, (b) RDE polarization curves, (c) K–L plots for ORR, (d) the number of transferred electrons and (e) Tafel plot for GO@100/GCE under oxygen saturated 0.1 M NaOH.

To further investigate the electrocatalytic activity of the rGO@100/GCE electrode towards the ORR, the experiments were carried out by rotating disc electrode (RDE) as indicated in Fig. 9. The RDE polarization (500 rpm) curves for the various electrodes are presented in Fig. 9a. The onset potential (*E*_on_) became less negative with the total dose. The *E*_on_ were −0.24, −0.21, −0.20, −0.16 and −0.15 V (*vs.* Ag|AgCl), for GO/GCE, rGO@25/GCE, rGO@50/GCE, rGO@75/GCE and rGO@100/GCE respectively. This observation suggests that increasing the total irradiation enhances the ORR activity of the electrode as per the observation in the CV studies. Congruent with the *E*_on_, the rGO@100/GCE electrode also exhibited the highest limiting current density (*J*_L_) (−0.87 mA cm^−2^) while the unirradiated GO recorded only −0.49 mA cm^−2^ at 500 rpm. Besides oxygen removal, the surface area of rGO@100/GCE was increased by more than six-fold and hence ORR active sites.^58^ The lower ORR activity of GO compared to rGO@100/GCE may be caused by excessive presence of oxygen in the catalyst which inhibited its ORR activity by lowering the turnover frequency of the reaction sites.^59^ The *E*_on_ for the rGO@100/GCE is comparable to and at times better than that of other rGO catalysts in literature.^56,60,61^ More literature is summarized in Table 5 to compare with the current study in (*vs.* RHE). Fig. 9b depicts that the rGO@100/GCE catalyst did not reach the diffusion-limited regime even at higher rotation rates. The polarization curve shows two regions; low potential region (*E* < −0.40 V) which is kinetically controlled while at higher potentials, (*E* > −0.4 to −0.8 V) the reaction is controlled by mixed kinetic-diffusion. The second wave at 0.53 V signals partial reduction of the peroxide formed by the 2-electron process. The number of electrons transferred (*n*) was determined by the K–L eqn (5)–(7).^62^5
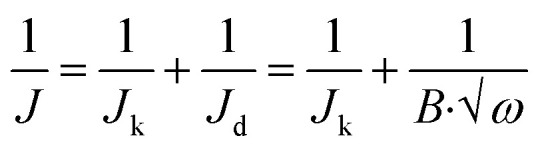

6 *B* = 0.62*FD*_0_^2/3^ν^−1/6^*C*_0_

7
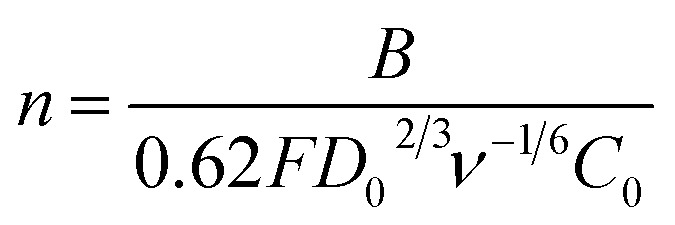


**Table 5:** Comparison of the as-synthesised rGO@100 ORR kinetics with other rGO catalysts in alkaline and neutral media in (V *vs.* RHE) using the rotating disk electrode

Material	Method/reducing agent	Synthesis conditions	Medium	*E*_on_ (*V*_RHE_)	Tafel slope (mV dec^−1^)	*J*_L_ (mA cm^2^) @−0.60 V and 1600 rpm	Average ‘*n*’	Ref.
rGO@100	Gamma irradiation/Avocado seed extract	RT/100 kGy	0.1 M NaOH	0.81	162	−1.17	2.04	This study
rGO@100	Gamma irradiation/Avocado seed extract	RT/100 kGy	0.1 M PBS	0.56	108	−2.32	2.26	This study
25% Fe Fe/N-rGO	Ethanol/Gamma irradiation	RT	0.1 M KOH	0.84	—	2.5	2.25	15
rGO	Hydrothermal/Avocado seed extract	100 °C	0.1 M NaOH	0.71	151	3.44	1.97	24
rGO	Thermal	900 °C under N_2_	0.1 M KOH	0.84	—	−4.54	3.41	56
rGO	CVD	—	0.1 M KOH	0.56	—	−0.17	—	60
N-rGO	CVD	—	0.1 M KOH	0.76	—	−0.8	3.6–4	60
rGO	Solvothermal/triethylenetetramine	—	0.1 M KOH	0.72	112	4.75	2.88	61
rGO	Chemical/hydrazine hydrate	100 °C	3.5% NaCl	0.94	—	3.2	3.5	64
rGO N700	Thermal	700 °C under N_2_	0.1 M KOH	0.80	70	−2.0	—	65
rGO HN500	Thermal	500 °C under H_2_ and N_2_	0.1 M KOH	0.82	76	−2.2	—	65
rGO sonic	Sonication	Ethanol and HCl	0.1 M KOH	0.85	92	−2.2	—	65

The slope (*B*) of the K–L plot was used to calculate the *n* occurring during ORR. In eqn (5), the current density (*J*) consists of a kinetic portion (*J*_k_) and a diffusion part (*J*_d_), *ω* is the angular velocity of the disk (*ω* = 2π*f*), *f* is number of revolutions per minute) and *B* is a constant that is related to *n*. In eqn (6), *F* is the Faraday constant (*F* = 96 485C mol^−1^), *D*_o_ is the diffusion coefficient of O_2_ in the 0.1 M NaOH electrolyte (1.90 × 10^−5^ cm^2^ s^−1^), *C*_o_ is the concentration of O_2_ in the 0.1 M NaOH solution (*C* = 1.38 × 10^−6^ mol L^−1^), *ν* = is the kinematic viscosity of the electrolyte (*ν* = 0.01 cm^2^ s^−1^). Fig. 9c shows linear and parallel K–L plots that are indicatives of a first order reaction controlled by kinetics and mass transport of oxygen species at the electrode surface. The parallel nature of the K–L plots suggests that the *n* values at the different potentials is very close to each other. The dependence of *n* on potential for the rGO@100/GCE is shown in Fig. 9d. In agreement with the parallel K–L plot, the *n* ranged between 1.98 and 2.07, very close to each other at different potentials. The average *n* was 2.04, which is a characteristic feature of an ORR catalyst that adheres to a 2-electron pathway. These results are consistent with the literature.^15,24,61^ However, some rGO catalysts exhibit higher *n* values as shown in Table 5 probably due to the differences in the synthesis procedures. The Tafel plot of the catalyst at 1600 rpm is presented in Fig. 9e. The slope was calculated to be −162 mV dec^−1^. The value is higher, an indication of slow reaction rate compared to reported modified rGO composites but is acceptable for nascent rGO.^63^ It is therefore believed that γ-ray irradiation of GO in the presence of *P. americana* Mill seed extract has the potential to yield robust rGO-based composite ORR catalysts.

#### 3.9.2. RDE studies in neutral conditions

**Fig. 10:**
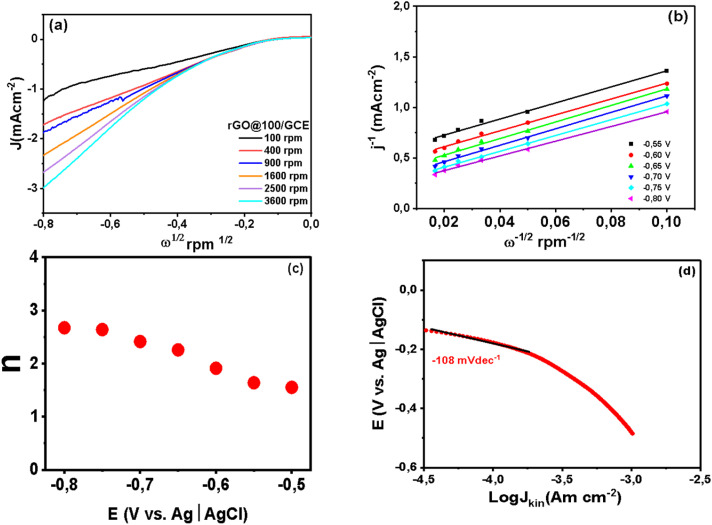
RDE polarization curves (a) gamma irradiated GO@100/GCE at various rotation rates at a scan rate of 5 mV (b) K–L plots for ORR, (c) the potential dependence of *n* as and (d) Tafel plot of GO@100/GCE in oxygen saturated 0.1 M PBS.

Given that ORR is also vital in neutral environments as it drives bioelectrochemical systems such microbial fuel cells (MFCs) and microbial desalination cells (MDCs), the performance of rGO@100 was also investigated in neutral medium. The medium was oxygen saturated 0.1 M PBS. A linear sweep voltammetry (LSV) from RDE test was performed with a scan rate of 5 mV s^−1^, rotation speed range of 100–3600 rpm in the potential range of −0.8 V to 0.1 V. The *E*_on_ was −0.08 V (*vs.* Ag|AgCl) or 0.56 V *vs.* RHE while the limiting current density at 0.8 V was −2.32 mA cm^−2^ at 1600 rpm (Fig. 10a). The absence of a well-defined diffusion-limited plateau signifies that even at high overpotential the current density is still in a mixed kinetic and mass transport-limited zone.^65^ It also implies that the ORR kinetics are too slow to reach a diffusion limited state in neutral media.^66^ The sluggish ORR in neutral media like PBS is due to low concentration of H^+^ and OH^−^ as well as low solubility and diffusion of oxygen gas compared to extreme pH environments.^67^ The K–L plot was constructed from 0.55–0.80 V (*vs.* Ag|AgCl) as shown in Fig. 10b where *D*_o_ = 2.1 × 10^−5^ cm^2^ s^−1^, *C*_o_ = 1.26 × 10^−6^ mol cm^−3^, and *ν* = 0.01 cm^2^ s^−1^. The linearity and parallelism of the K–L plots fitting lines insinuates first-order reaction kinetics with respect to dissolved O_2_. The *n* value increased from 1.55 at −0.55 V to 2.67 at −0.8 V (Fig. 10c) averaging at 2.26. It is apparent that *n* is dependent on the applied potential indicating that rGO@100 possibly catalyzes the reaction in more than one reaction pathways in this medium.^68^Fig. 10d shows a Tafel slope that was calculated to be 108 mV dec^−1^.

## 4. Conclusions

Graphene oxide dissolved in *P. americana* Mill seed extract was successfully reduced *via* γ-irradiation technique at a total dose of 100 kGy in the absence of an inert atmosphere. The reduction was confirmed by the UV-Vis, FTIR, TEM and Raman spectroscopy. The XRD revealed a slow reduction and thus remains of oxygenated groups of graphene oxide probably due to the absence of inert environment. The reduction proved that the present natural antioxidants in *P. americana* Mill seed extract can replace synthetic aprotic solvents when the γ-ray irradiation method is employed to reduce GO. The BET revealed that irradiation enhanced the *S*_BET_ and pore volume of rGO@100 approximately 6 times compared to that of GO. The enhancement in the surface area was evidenced by the high current response of the corresponding electrode in the CV and EIS studies. The *R*_ct_ was reduced from 36.1 kΩ for GO to 1.07 kΩ for rGO@100 which is an indication of increased conductivity with reduction. Based on the CV for ORR studies, the rGO@100/GCE electrode in an alkaline and neutral media adhered to a diffusion-limited electrochemical process. According to K–L plots the *n* suggests that the as-formed electrode works better in PBS which is mostly employed as catholytes in MFCs and MDCs. Since unmodified rGO@100/GCE follows the 2e^−^ reaction pathway which is also the reported result when other synthesis methods are used, the combination of plant extract and γ-irradiation method in the absence of nitrogen gas has the potential to yield robust catalyst upon modifications. Therefore, the efficiency of the synthesis method is worth exploring towards the synthesis of modified rGO/graphene composites for the ORR application.

## Author contributions

Nkosingiphile E. Zikalala: writing – original draft, conceptualization, review & editing. Shohreh Azizi: funding acquisition, supervision, conceptualization, writing – review & editing. Ali. A. Zinatizadeh: supervision, conceptualization, visualization. Nomvano Mketo: supervision, writing – review & editing. Touhami Mokrani: writing – review & editing. Malik M. Maaza: supervision, writing – review & editing.

## Conflicts of interest

The authors declare that there are no known financial and personal competing interests.

## Supplementary Material

NA-008-D5NA01150G-s001

## Data Availability

All data supporting the findings of this study are available within the article. Additional data are contained in the supplementary information (SI), where Table S1 summarises additional data from the BET studies, section S1 and Fig. S1 are a discussion on the Bode plots, and expansion of the EIS discussion. Supplementary information (SI) is available. See DOI: https://doi.org/10.1039/d5na01150g.
