# Supplementary material for: Gamma radiation assisted reduction of graphene oxide in Persea americana Mill. seed extract: characterization and oxygen reduction reaction in alkaline and neutral media
Source: Nanoscale Adv. 2026 Mar 10;8(7):2411–25. doi: 10.1039/d5na01150g (PMC12973225; doi:10.1039/d5na01150g)
Supplement: NA-008-D5NA01150G-s001 [file NA-008-D5NA01150G-s001.pdf]

Supplementary Information (SI) for Nanoscale Advances.
This journal is © The Royal Society of Chemistry 2026

Supplementary information

Gamma radiation assisted reduction of graphene oxide in *Persea americana Mill.* seed extract: characterization and oxygen reduction reaction in alkaline and neutral media

Nkosingiphile E. Zikalala [a,b], Shohreh Azizi [a*], Nomvano Mketo [b], Ali. A. Zinatizadeh [c], Touhami Mokrani [d], Malik M. Maaza [a,e]

[a] *UNESCO-UNISA Africa Chair in Nanosciences & Nanotechnology Laboratory, College of Graduate Studies, University of South Africa, Muckleneuk Ridge, P.O. Box 392, Pretoria 0002, South Africa*

[b] *Department of Chemistry, College of Science and Engineering and Technology, Florida Science Campus, University of South Africa, Johannesburg, South Africa*

[c.] *Environmental Research Canter, Department of Applied Chemistry, Razi University, Kermanshah P.O. Box 67144-14971, Iran*

[d.] *Department of Chemical Engineering, University of South Africa, Private Bag X6, Florida, Johannesburg 1709, South Africae*

[e.] *Nanosciences African Network (NANOAFNET)-Materials Research Department, iThemba LABS-National Research Foundation, Somerset West, P.O. Box 722, Cape Town 7129, South Africa*

Corresponding author: azizis@unisa.ac.za (Shohreh Azizi)

Table S1: Surface area analysis of GO and rGO@100

**Property**	**GO**	**rGO@100**
Surface area (m^2/g), BET	9.683	60.345
Pore volume (cm^3/g), BJH ads	0.020	0.076
Pore diameter (nm), BJH ads	18.898	7.195
Pore volume (cm^3/g), BJH des	0.022	0.076
Pore diameter (nm), BJH des	13.779	5.504
Isotherm type	4	4

S1.

Bode plots contain key information corresponding to the applied frequency such as increased sensitivity to changes in capacitance, resistance, and ion diffusion.[1] Bode phase angle plots for the electrodes in Figure S2a show that the electrodes exhibited capacitive relaxation at different phase angles and frequency from one to another. This indicates that the electrodes experience different charge transfer kinetics at the solution-electrode interface from one electrode to the other.[2] The electrodes exhibit lack of an ideal capacitive behavior since capacitors phase angle is -90° at low frequences and approaches zero at high frequences.[3] According to Muthurasu et al. the higher phase angle values even at lower frequencies, where the ionic permeation is favored, point to the fact that these catalyst form highly compact monolayers with very few defects on the electrode surface.[4] At lower frequencies (0.001 f/kHz) the phase angle for rGO@75/GCE is -35° and increases to -50° (0.01 f/kHz) before declining to -10° at 10 f/kHz. This implies that this electrode is more affected by ionic resistance than the other electrodes. This observation corroborates the higher R_{ct} value it exhibits in the Nyquist plots compared to rGO@50/GCE and rGO@100/GCE respectively. The impedance of the CPE (Z_{CPE}) is a power-law-dependent interfacial capacity as shown in Equation S1: where Q is the frequency-independent constant, ω is the radial frequency, and the exponent n arises from the slope of log Z vs. log f. The Bode magnitude plots for the various electrodes are shown in Figure S2b where f is the frequency. An n value of 0 corresponds to a pure resistor; n = 1 corresponds to a pure capacitor, while a 0.5 n value corresponds to Warburg impedance.[2] The values of the slope at mid frequencies for the respective electrodes are GCE (−0.81), GO/GCE (-0.78), rGO@25/GCE (-0.77), rGO@50/GCE (-0.73), rGO@75/GCE (-0.71), rGO@100/GCE (-0.78). These values are less than -1.0 indicating that these electrodes exhibit pseudo-capacitive behavior as also suggested by the Bode plots. The pseudo capacitive nature of the electrode (rGO@100/GCE) makes it a promising candidate for catalytic applications and storage devices.

$$Z_{CPE} = \frac{1}{\left[Q(jw)^n\right]} \quad \text{(S1)}$$

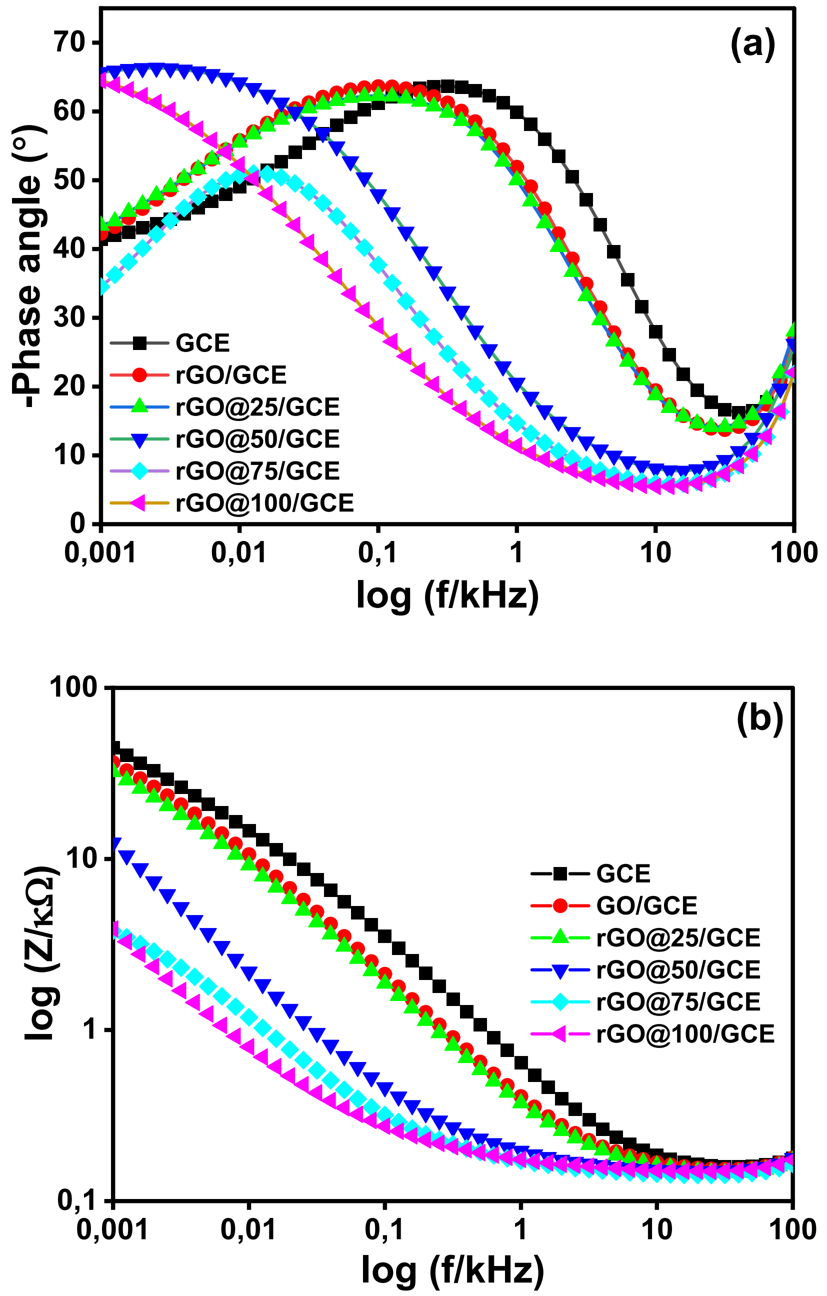

Figure S1. (a) Bode phase angle plots (b) Bode magnitude plots obtained for the various electrodes studied in 0.1 M KCl containing 1 mM $[Fe(CN)_6]^{3-/4-}$

References

[1] N.O. Laschuk, E.B. Easton, O. V Zenkina, Reducing the resistance for the use of electrochemical impedance spectroscopy analysis in materials chemistry, RSC Adv. 11 (2021) 27925–27936. https://doi.org/10.1039/d1ra03785d.

[2] B.O. Agboola, S.L. Vilakazi, K.I. Ozoemena, Electrochemistry at cobalt (II) tetrasulfophthalocyanine- multi-walled carbon nanotubes modified glassy carbon electrode : a

sensing platform for efficient suppression of ascorbic acid in the presence of epinephrine, J Solid State Electrochem. 13 (2009) 1367–1379. https://doi.org/10.1007/s10008-008-0691-3.

[3] M.S. Almanto, Electrochemical and electrocatalytic properties of iron(II) and cobalt(II) phthalocyanine complexes integrated with multi-walled carbon nanotubes, University of Pretoria, 2010.

[4] A. Muthurasu, V. Ganesh, Electrochemical characterization of Self-assembled Monolayers (SAMs) of silanes on indium tin oxide (ITO) electrodes – Tuning electron transfer behaviour across electrode – electrolyte interface, J. Colloid Interface Sci. 374 (2012) 241–249. https://doi.org/10.1016/j.jcis.2012.02.007.
